# TAK-715 alleviated IL-1β-induced apoptosis and ECM degradation in nucleus pulposus cells and attenuated intervertebral disc degeneration ex vivo and in vivo

**DOI:** 10.1186/s13075-023-03028-4

**Published:** 2023-03-21

**Authors:** Kun Wang, Dengbo Yao, Yuxi Li, Ming Li, Weike Zeng, Zhuangyao Liao, Engming Chen, Shixin Lu, Kaihui Su, Zhen Che, Yuwei Liang, Peng Wang, Lin Huang

**Affiliations:** 1 Department of Orthopedics, Sun Yat-Sen Memorial Hospital, Sun Yat-Sen University, 107 Yanjiang West Road, Guangzhou, 510120 China; 2 Department of Orthopedics, Eighth Affiliated Hospital of Sun Yat-Sen University, Sun Yat-Sen University, 3025 Shennan Middle Road, Shenzhen, 518033 China; 3 Department of Radiology, Sun Yat-Sen Memorial Hospital, Sun Yat-Sen University, Guangzhou, China; 4 Department of Orthopedics, Nangchang First Hospital, Nanchang, China

**Keywords:** TAK-715, Intervertebral disc degeneration, IDD, Inflammation, Apoptosis, p38MAPK

## Abstract

**Background:**

Intervertebral disc degeneration (IDD) is one of the most common disorders related to the spine. Inflammation, apoptosis and extracellular matrix (ECM) degradation contribute to disc degeneration in nucleus pulposus cells (NPCs). This study focused on the role and mechanism of the p38 inhibitor TAK-715 in intervertebral disc degeneration.

**Methods:**

NPCs were treated with IL-1β to mimic apoptosis, followed by the addition of TAK-715. It was determined that apoptosis, inflammatory mediators (COX-2), inflammatory cytokines (HMGB1), and ECM components (collagen II, MMP9, ADAMTS5, and MMP3) existed in NPCs. In addition, the p38MAPK signaling pathways were examined. The role of TAK-715 in vivo was determined by acupuncture-induced intervertebral disc degeneration. Following an intradiscal injection of TAK-715, MRI and a histopathological analysis were conducted to assess the degree of degeneration.

**Results:**

IL-1β-induced apoptosis was alleviated by TAK-715 in vitro, and antiapoptotic proteins were upregulated. Furthermore, TAK-715 blocked IL-1β-induced inflammatory mediator production (COX-2) and inflammatory cytokine production (HMGB1) and degraded the ECM (collagen II, MMP9, ADAMTS5, and MMP3). By inhibiting the phosphorylation of p38, TAK-715 exerted its effects. In a rat tail model, TAK-715 ameliorates puncture-induced disc degeneration based on MRI and histopathology evaluations.

**Conclusion:**

TAK-715 attenuated intervertebral disc degeneration in vitro and in vivo, suggesting that it might be an effective treatment for IDD.

## Introduction

Low back pain (LBP) is a common symptom of intervertebral disc degeneration (IDD), with an 80% lifetime prevalence [1]. In addition, approximately 10% of those impacted are chronically ill and incapacitated, placing significant economic strain on the entire world [1, 2]. The intervertebral disc (IVD), a composite tissue, is comprises the following two parts: an inner nucleus pulposus (NP) and an outer annulus fibrosus. In maintaining homeostasis, Nucleus pulposus cells (NPCs) secrete a complex extracellular matrix (ECM), which consists mainly of collagen II. This ECM is vital for maintaining the physiological viscoelasticity of IVDs [3]. IDD is characterized by cellular loss, ECM degradation, and spinal flexibility loss [4]. Human nucleus pulposus cells (HNPCs) secrete proinflammatory molecules that may promote ECM-degrading enzymes in intervertebral disc degeneration. However, the degeneration mechanisms underlying IDD remain unclear.

The nucleus pulposus is composed of NPCs, which are responsible for disc degeneration when they undergo reduction. Nuclear pulposus cells are susceptible to oxidative stress, cellular senescence, inflammation, apoptosis, etc. As a leading cause of IDD, inflammation and apoptosis have been identified [5, 6]. An increase in the expression of IL-1β was observed in NP tissue as disc degeneration progressed. IL-1β induces multiple inflammatory mediators in NPCs, including cyclooxygenase-2 (COX-2) and inducible nitric oxide synthase (iNOS) [7]. And IL-1β increases the level of inflammatory cytokines, such as high mobility group protein 1 (HMGB1) [8]. When IL-1β is upregulated, ECM degradation is attributed to matrix metalloproteinases (MMPs) and a disintegrin and metalloproteinase with thrombospondin motifs (ADAMTS). In addition, increased IL-1β induces NPC apoptosis, causing leakage of NPCs and reduced ECM synthesis. The occurrence of apoptosis and ECM degradation in NPCs is associated with the activation of the p38MAPK pathway [9]. Therefore, these findings suggest that IDD is closely related to apoptosis.

Apoptosis is regulated by a variety of pathways, including p38 MAPK, NF-κB, and PI3K/AKT. The activation of p38MAPK is thought to be one of the mechanisms responsible for human intervertebral disc degeneration [10, 11]. When p38MAPK is activated, inflammatory factors can aggregate and contribute to the degeneration of IVD. Then, apoptosis is triggered, and tissue metalloproteinases are released, further progressing spinal degenerative diseases. TAK-715, as a potent inhibitor of p38 mitogen-activated protein kinase, has shown therapeutic effects in inflammatory diseases. A rat arthritis model has shown that TAK-715 exerts anti-inflammatory effects via p38MAPK [12]. However, the effect of TAK-715 on intervertebral discs via p38MAPK is unknown. TAK-715 may be a promising IDD treatment since it inhibits p38MAPK.

The aim of our research was to investigate the anti-inflammatory and antiapoptotic effects of TAK-715 on IL-1β-stimulated NPCs and explore the possible mechanism of action. Through the inhibition of p38 MAPK signaling, TAK-715 ameliorated inflammation and apoptosis. Furthermore, TAK-715 exerted protective effects ex vivo and in vivo against IDD induced by puncture. As a result of this work, we conclude that TAK-715 may be a promising treatment target for IDD.

## Methods

### Cell culture

Rat NPCs were provided as a gift by Dr. Chen Di of Rush University Medical Center (Chicago, IL, USA) [13]. After SD rats (male, weight 200–250 g) were euthanized, NP tissues were harvested from caudal intervertebral discs, and then digested in 0.2% pronase for 1 h at 37 °C, followed by 2.5% collagenase II digestion for 15 min at 37 °C. Then, the digested tissues were washed and cultured in Dulbecco's modified Eagle’s medium (DMEM) containing 10% fetal bovine serum (FBS) and 1% penicillin–streptomycin at 37 °C. The NP cells were cultured in DMEM with 5% CO_2_ at 37 °C. We replaced medium every two days and passaged until they reached 80–90% confluence [1, 2].

### Cell viability analysis

Ninety-six-well plates were seeded with 4 × 10^3^ cells/well for 24 h before TAK-715 (0, 0.05, 0.1, 0.25, 0.5, 1.0, 5 and 10 μM in PBS; Med Chem Express, MCE, China) was added at increasing concentrations for 24 and 48 h. CCK-8 (Med Chem Express, MCE, China) was used to assess the cell viability following treatment with TAK-715. We cultured the cells at 37 °C for one hour in 10 μL of CCK-8 reagent in fresh intact medium. The simulated control was untreated cells, while the blank control was intact medium containing CCK-8 reagent. The measurement of absorbance at 450 nm was conducted with an optical density reader.

### Quantitative real-time polymerase chain reaction

Using EZB reagent (MA, USA), the total RNA was extracted from NPCs and analyzed using real-time quantitative PCR (qPCR). Prime Script RT Master Mix was used to transcribe complementary DNA (cDNA) from the extracted RNA. SYBR Premix Ex Taq was used for real-time qPCR. The primers for collagen II (forward: GCCAGGATGCCCGAAAATTAG reverse: CTCGTCAAATCCTCCAGCCA); MMP9 (forward: GATCCCCAGAGCGTTACTCG, reverse: GTTGTGGAAACTCACACGCC); ADAMTS5 (forward: AAAACTGGCGAGTACCTT, reverse: TCCTTTGTGGCTGAATAG); and MMP3 (forward: ACCCAGCCCTATCCCTTGAT, reverse: TCTCGGGATGGATGCTCGTA) were described in a previous study, and GAPDH (forward: GGCACAGTCAAGGCTGAGAATG reverse: GGTGGTGAAGACGCCAGTA) was used for normalization. The △△CT method was used to determine the expression of the target mRNAs.

### Western blot analysis

Proteinase inhibitor (1%) and phosphotransferase inhibitor (1%) (Cwbio, Jiangsu, China) were added to RIPA buffer before the NPC samples were lysed. Proteinase lysis was followed by centrifugation and total protein extraction. Finally, each sample was subjected to a western blot analysis using 20 μg of protein. As a result of different weights, the preboiled samples of protein along with loading buffer were electrophoresed for 90 min on a 10% or 12% SDS‒PAGE gel. Then, the gel particles were transferred to PVDF membranes (Millipore, Billerica, MA, USA).

The following antibodies were used: Abcam: anti-collagen II (1:1000), anti-MMP3 (1:1000), anti-HMGB1 (1:1000), anti-MMP9 (1:1000) and anti-ADAMTS5 (1:1000); CST: anti-p38 (1:1000) and anti-p-p38 (1:1000); Immunoway:anti-Bcl2 (1:1000), anti-Bax (1:1000) and anti-Cleaved caspase3(1:1000); Huabio: anti-Cox2 (1:1000); and Cwbio: anti-GAPDH (1:5000) and anti-Tubulin (1:5000).

TBST was used to wash the membranes after incubation with the primary antibodies, followed by one hour of incubation with special secondary antibodies. Then, the membranes were incubated with specific primary antibodies overnight at 4 °C, followed by blocking in TBST solution with 5% BSA for 1 h. Then, the membranes were incubated with secondary antibodies for one hour after washing three times. The final analysis was performed using an ECL imager (Syngene G: BOX ChemiXT4, United Kingdom) to detect and analyze the signals.

### Immunofluorescence

The cells were washed three times with PBS before being fixed with 4% paraformaldehyde and permeabilized with 0.5% Triton X-100 for 5 min. Bovine serum albumin (10%) was applied for one hour at 37 °C to block nonspecific protein binding. Then, PBS was applied, and primary antibodies against MMP-9 (1:200), Cox-2 (1:100) and p-p38 (1:100) were applied in a humid chamber overnight at 4 °C. Following the incubation with the primary antibodies, the cells were washed again with PBS and incubated for 1 h at 37 °C with the secondary antibody (Alexa Fluor® 488/594 conjugated, 1:100). Fluorescence images were taken under an Olympus BX63 microscope (NY, USA) after three rounds of washing with PBS and staining with DAPI.

### Surgical procedures

Sun Yat-Sen University's Institutional Animal Care and Use Committee approved all animal experiments (SYSU-2022-G0104). Sprague–Dawley rats (200–250 g) were obtained from Sun Yat-sen University's Laboratory Animal Center and randomly assigned to one of three groups, including the control group (*n* = 6), IDD group (PBS injected after surgery, *n* = 6) and TAK-715 group (TAK-715 injected after surgery, *n* = 6). The rats in the IDD group (50 mg/kg) and TAK-715 group were anesthetized with 2% pentobarbital (50 mg/kg), and the entire annulus fibrosus layer was punctured with a needle (21G) through the tail skin. All needles were kept in the discs for 1 min. A dose of 5 μl (1 μM) of TAK-715 was immediately injected intradiscal into the TAK-715 group, and the same amount of PBS was injected into the IDD group after surgery. Before and after surgery, a pathogen-free environment was provided for all rats in addition to free food and water access. In a temperature-controlled room, the animals were kept on a 12-h light/dark cycle.

### Effect of TAK-715 in an ex vivo IVD culture model

We collected IVDs with complete end plates from rats. Then, we used DMEM containing 10% FBS and 1% penicillin/streptomycin to culture IVDs. A previous study suggested that the culture medium should be osmolar (400 mOsm) [14]. The samples were kept at 37 °C with 5% oxygen and saturated humidity during incubation. IVDs were treated with TAK-715. During the culture process, the medium was changed every two days.

### Magnetic resonance imaging method

The disc degeneration of the rats was examined by MRI eight weeks after surgery. All rats were injected with an overdose of pentobarbital (50 mg/kg) intraperitoneally for euthanasia. Using a 3.0 T clinical magnet (Philips Intera Achieva 3.0MR), we evaluated the signals and structures of all rats’ tails by magnetic resonance imaging in sagittal T2-weighted images [15]. An evaluation of the IDD degree was carried out according to the Pfirrmann system [16].

### Histological and immunohistochemical (IHC) staining

The specimens were fixed in 4% paraformaldehyde for 48 h, decalcified for over 30 days, and embedded in paraffin. For the assessment of disc degeneration, sections of the sample were stained with HE and Safranin O-fast green. Incubation with primary antibodies against p-p38 (1:200), collagen II (1:200) and BCL2 (1:200) followed by secondary antibodies was applied to the rat disc samples. The histology scores were calculated according to a grading system [17, 18]. ImageJ software was used to visualize the images. The antibodies were purchased from Immunoway (Plano, USA).

### Statistical analysis

Each experiment was repeated at least three times. The results are expressed as the mean ± S.D. The statistical analyses were conducted using GraphPad Prism 8 software (La Jolla, CA, USA). ANOVA was used to compare the control and treatment groups, followed by Tukey's test or t test (and nonparametric tests). This study used Kruskal‒Wallis H tests to analyze the nonparametric data (Pfirrmann grading). *P* < 0.05 was used to establish statistical significance.

## Results

### Effects of TAK-715 on NPC viability

**Fig. 1:**
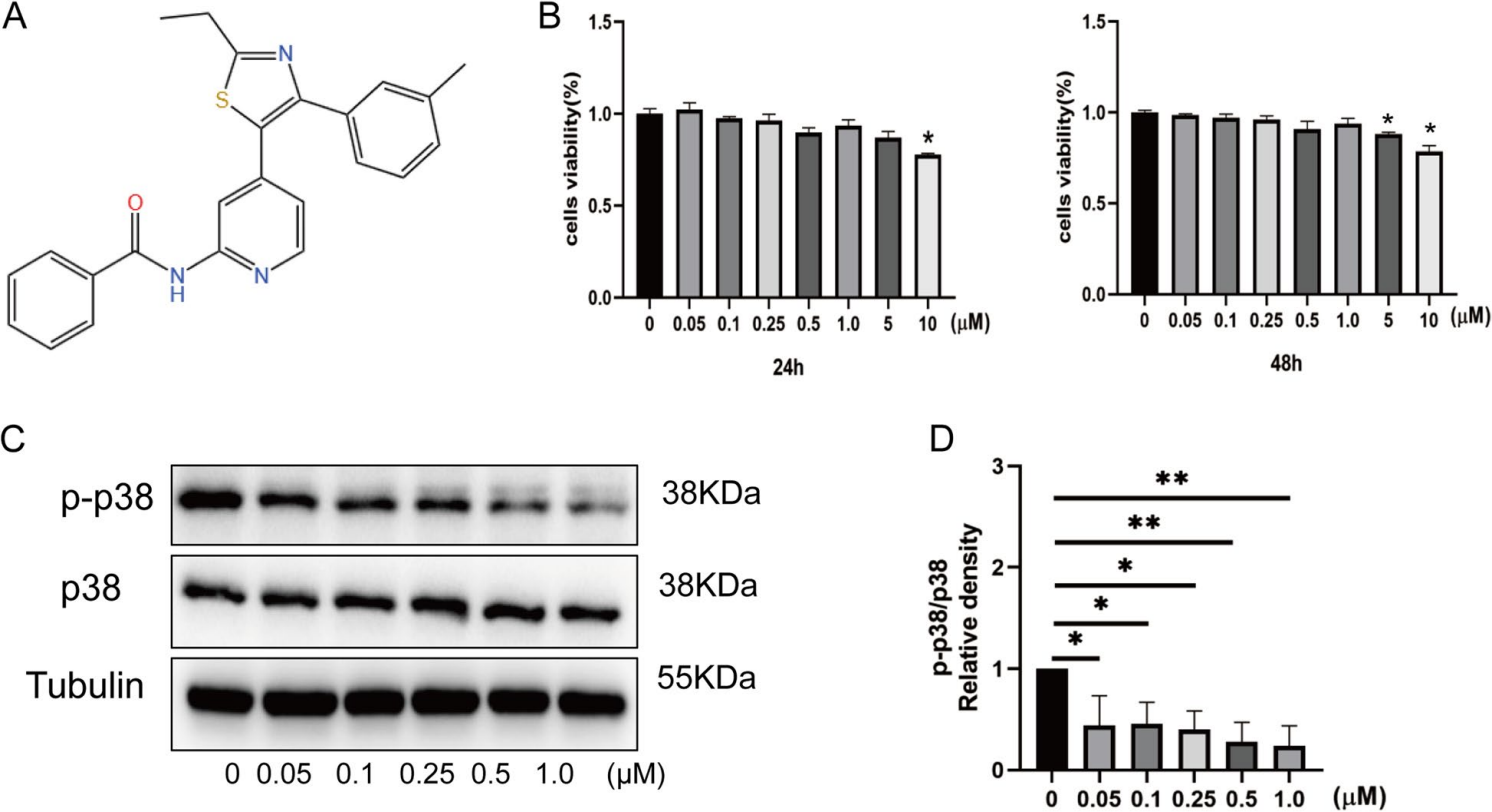
TAK-715 effects on NPC viability. **A** Chemical structure of TAK-715. **B** A CCK8 assay was used to determine the cytotoxic effects of TAK-715 on NP cells for 24 h and 48 h. **C** Western blot analysis was used to analyze p38 and p-p38 protein expression in NPCs treated with various concentrations of TAK-715; **D** the immunoblot ratio of p-p38/p38 was quantitatively analyzed. The data are presented as the mean ± SD (*P < 0.05; **P < 0.01; ***P < 0.001)

The chemical structure of TAK-715 is shown in Fig. 1A. To evaluate whether TAK-715 has toxic effects on NPCs, NPCs were treated with TAK-715 for 24 and 48 h. A CCK8 assay was then performed using NPCs to determine their viability. Figure 1B show that TAK-715 was not toxic to NPCs at concentrations below 1.0 μM and that TAK-715 could slightly decrease the cellular activity of NPCs. Additionally, TAK-715 suppressed the expression of p-p38MAPK up to 1.0 μM dose-dependently (Fig. 1C, D). Therefore, the TAK-715 concentration applied in the subsequent experiments was below 1.0 μM.

### TAK-715 alleviates IL-1β-induced ECM degradation in NPCs

**Fig. 2:**
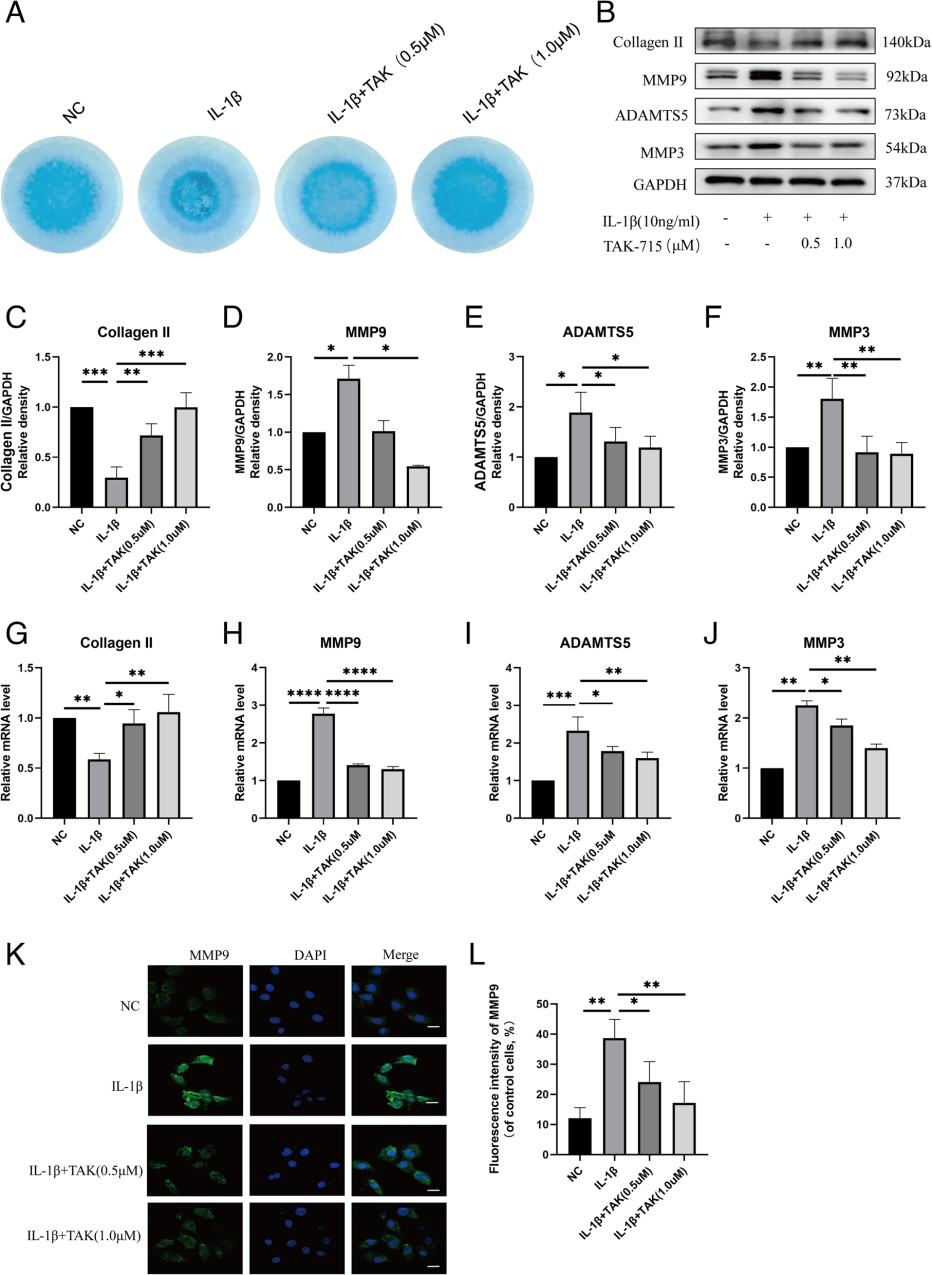
TAK-715 suppressed IL-1β-induced ECM degradation in NPCs. **A** NP cells were seeded in 24-well plates at 10.^7^/ml, which were in a 2D system. NP cells were co-treated with TAK-715 and IL-1β for 5 days, and then stained with alcian blue. **B** Western blot analysis was used to analyze Collagen II, MMP9, ADAMTS5 and MMP3 in NPCs; **C**-**F** The immunoblots of Collagen II, MMP9, ADAMTS5 and MMP3 were quantitatively analyzed. **G**-**J** The mRNA expression of Collagen II, MMP9, ADAMTS5 and MMP3 was measured by qRT‒PCR. **K** The fluorescence intensity of MMP9 was analyzed by immunofluorescence assays (scale bar:20 μm). **J** ImageJ was used to analyze the fluorescence intensity of MMP9. The data are presented as the mean ± SD (*P < 0.05; **P < 0.01; ***P < 0.001; ****P < 0.0001)

To detect the function of TAK-715 in NPCs, a high-density NPC culture model was used to simulate NPC degeneration stimulated by inflammatory factors in vitro. IL-1β stimulates collagen degradation in NPCs (Fig. 2A). A significant decrease in the ratio of collagen degradation was observed in the NPCs in response to IL-1β compared with the treatment group. The intervertebral disc mainly comprises the ECM, and the degeneration of the ECM is known to accelerate disc degeneration. A further test was conducted to determine whether TAK-715 inhibited the degradation of the ECM. By conducting western blot and real-time PCR analyses, we evaluated the expression of collagen II (a major component of the ECM), MMPs and ADAMTS5 in NPCs. After the IL-1β treatment, the NPCs expressed a decreased level of collagen II, whereas MMP9, ADAMTS5 and MMP3 were upregulated (Fig. 2B-F). The NPCs showed reduction of collagen II and increase of MMP9, ADAMTS5 and MMP3 in the mRNA expression after the IL-1β treatment. Nevertheless, the mRNA expression of Collagen II, MMP9, ADAMTS5 and MMP3 in NP cells was reversed after the TAK-715 treatment in a dose-dependent manner (Fig. 2G-J). Moreover, we determined MMP9 expression using immunofluorescence staining, and a specific antibody was used to label MMP9. As shown in Fig. 2K, L, the MMP9 fluorescence intensities were increased in the NPCs after the IL-1β stimulation, but this phenomenon was reversed when TAK-715 was added. Consequently, TAK-715 inhibited IL-1β-induced ECM degradation in NPCs.

### TAK-715 inhibits the generation of COX-2 and HMGB1 in IL-1β-stimulated NPCs

**Fig. 3:**
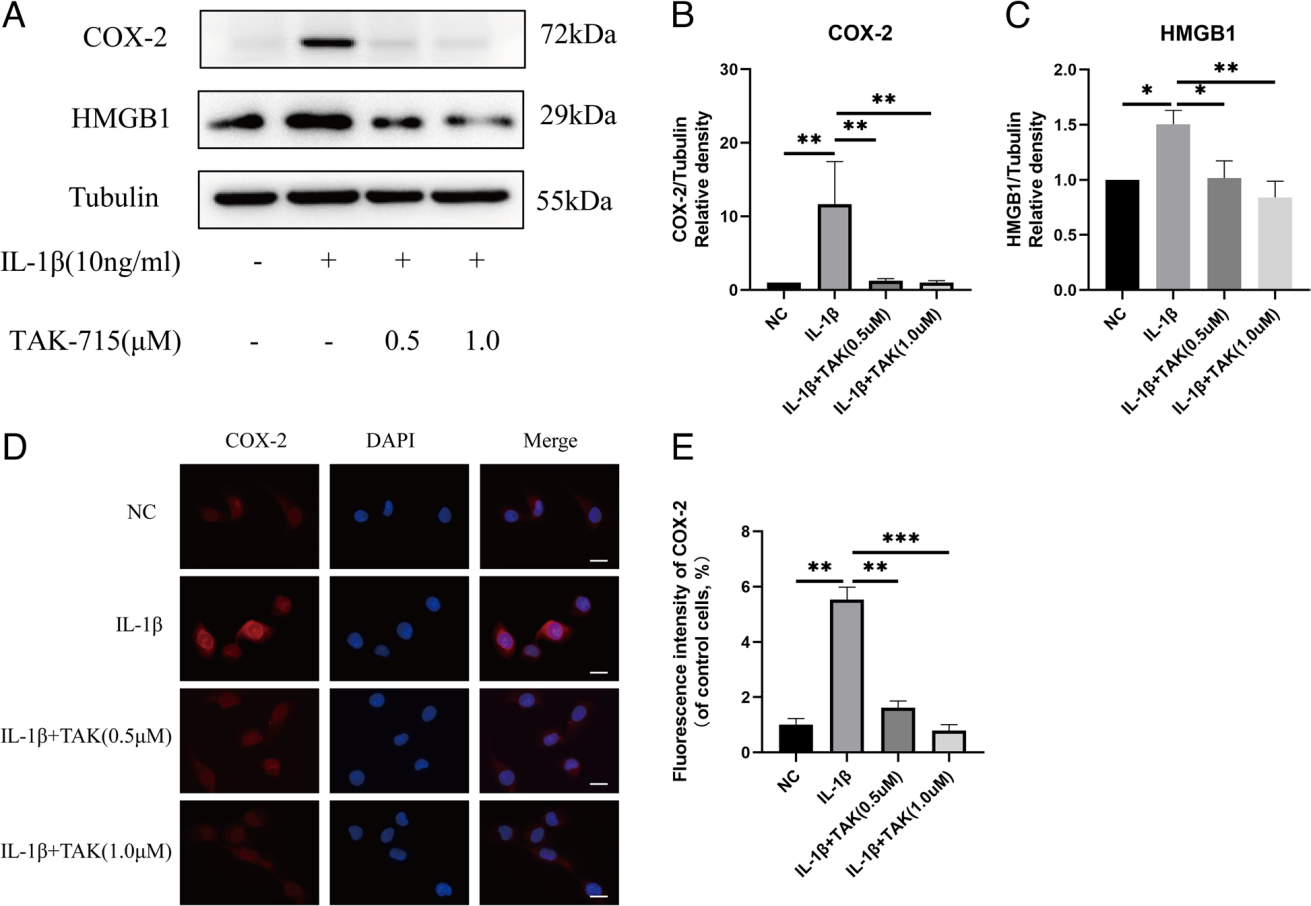
TAK-715 alleviated IL-1β-induced inflammation in NPCs. NPCs were preincubated with TAK-715 for 2 h, and then, IL-1β was added for 48 h. **A** Western blot analysis was used to analyze COX-2 and HMGB1 in NPCs; **B**, **C** COX-2 and HMGB1 immunoblots were quantitatively analyzed. **D** The fluorescence intensity of COX-2 was analyzed by immunofluorescence assays (scale bar: 20 μm). **E** ImageJ was used to analyze the fluorescence intensity of COX-2. The data are presented as the mean ± SD (*P < 0.05; **P < 0.01; ***P < 0.001)

A western blot analysis was used to determine the effects of TAK-715 in NPCs under inflammatory conditions induced by IL-1β. Under IL-1β stimulation, the NPCs expressed significantly higher levels of COX-2 and HMGB1 (Fig. 3A-C). Nevertheless, TAK-715 dose-dependently inhibited this increase. In addition, we determined COX-2 expression using immunofluorescence staining (Fig. 3D, E). The fluorescence intensities of COX-2 were increased in the NPCs after the IL-1β stimulation, but TAK-715 inhibited the increase. According to these results, TAK-715 significantly inhibited IL-1β-induced inflammatory mediator and cytokine production.

### TAK-715 protects nucleus pulposus cells from IL-1β-induced apoptosis

**Fig. 4:**
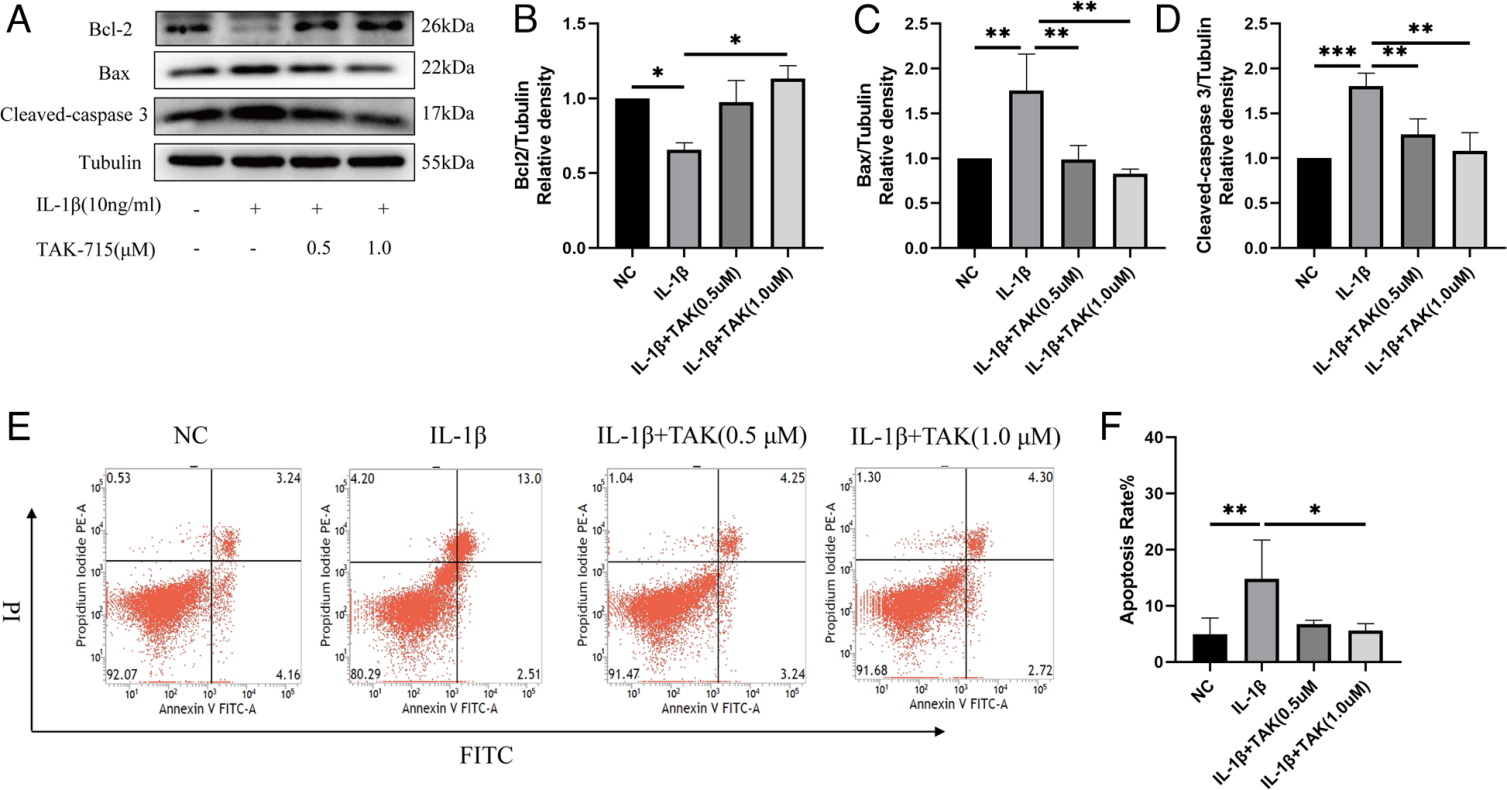
TAK-715 reduced IL-1β-induced apoptosis in NPCs. NPCs were preincubated with TAK-715 for 2 h, and then, IL-1β was added for 48 h. **A** Western blot analysis was used to analyze Bcl-2, Bax, and cleaved caspase-3 in NPCs; **B**-**D** Immunoblots of Bcl-2, Bax, and cleaved caspase-3 were quantitatively analyzed. **E** Flow cytometric measurement of the apoptosis incidence in NPCs. **E** The apoptosis rate was quantitatively analyzed. The data are presented as the mean ± SD (*P < 0.05; **P < 0.01; ***P < 0.001)

As COX-2 and HMGB1 play an important role in cell apoptosis, we investigated whether TAK-715 affected NP cell apoptosis in our study since apoptosis contributes to IDD progression. According to the western blot analysis (Fig. 4A-D), Bax and cleaved-caspase 3 were increased in the NPCs by IL-1β, while Bcl-2 was decreased. In contrast, a dose-dependent increase in Bcl-2 and decrease in Bax and cleaved-caspase 3 expression was observed in the NPCs induced by IL-1β in response to TAK-715. Additionally, as a result of flow cytometry (Fig. 4E, F), IL-1β increased apoptosis in the NPCs, while TAK-715 prevented this increase in the NPCs. It was concluded that TAK-715 reduces apoptosis in NPCs after IL-1β stimulation in vitro.

### TAK-715 inhibits p38MAPK pathway activation in IL-1β-treated NPCs

**Fig. 5:**
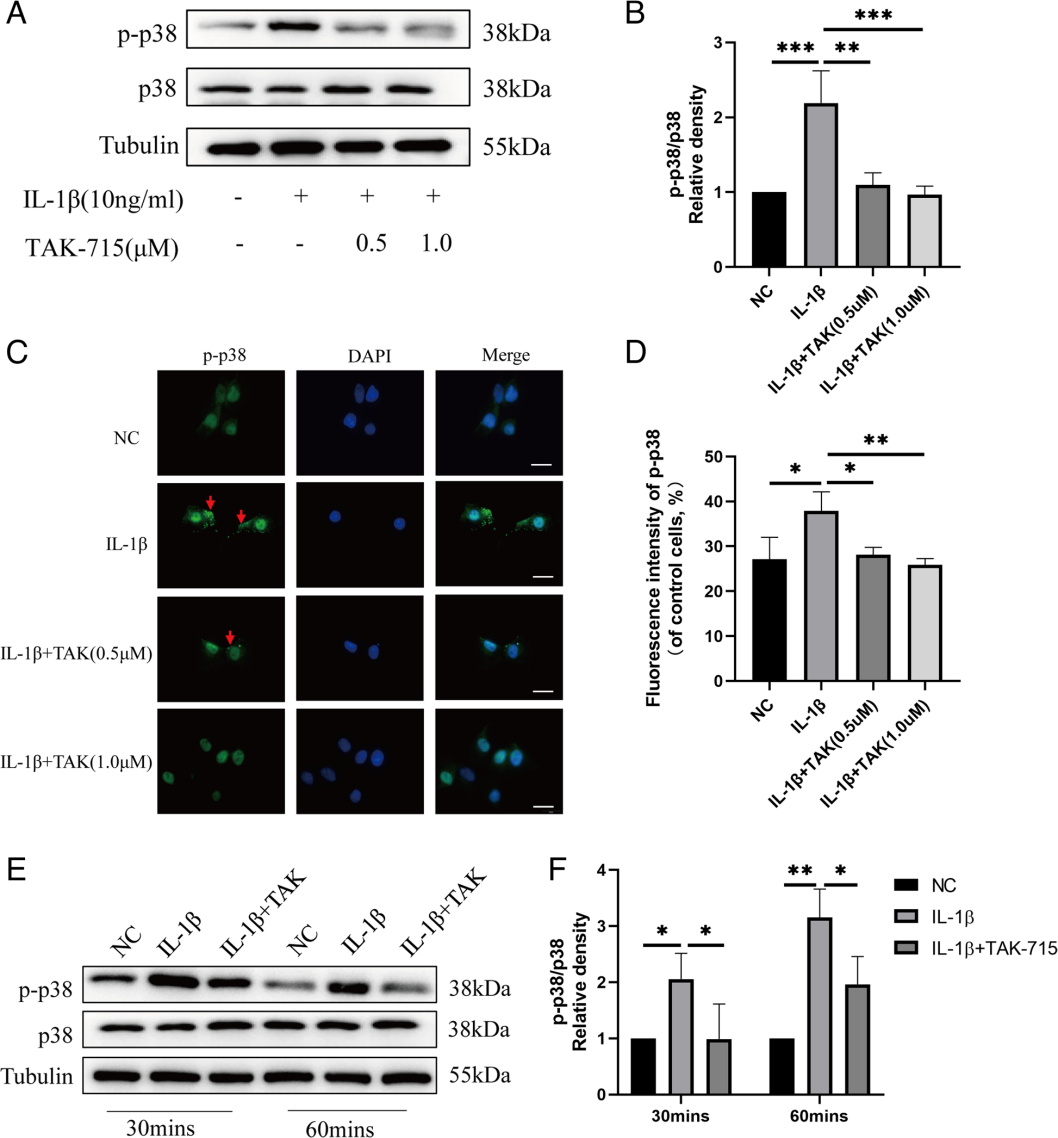
TAK-715 suppressed IL-1β-induced p38 activation in NPCs. NPCs were preincubated with TAK-715 for 2 h, and then, IL-1β was added for 48 h. **A** Western blot analysis was used to analyze p38 and p-p38 protein expression in NPCs. **B** The immunoblot ratio of p-p38/p38 was quantitatively analyzed. **C** The fluorescence intensity of p-p38 was analyzed by immunofluorescence assays (scale bar:20 μm). **D** ImageJ was used to analyze the fluorescence intensity of p-p38. Western blot analysis was used to detect the expression of p38 and p-p38 protein in NPCs treated with IL-1β at 30 min and 60 min, respectively. The data are presented as the mean ± SD (*P < 0.05; **P < 0.01; ***P < 0.001)

This study aims to investigate the possible mechanism by which TAK-715 inhibits inflammation in NPCs. NPCs were preincubated with TAK-715 (0.5 μM and 1.0 μM) for 2 h, and co-treated with TAK-715 and IL-1β (10 ng/ml) for 48 h. IL-1β enhanced p-p38 expression in NPCs, but TAK-715 decreased p-p38 expression in a dose-dependent manner (Fig. 5A, B). Additionally, the immunofluorescence staining of p-p38 in NPCs showed that IL-1β increased the phosphorylation of p38 in both the cytoplasm (red arrow) and nucleus, whereas TAK-715 decreased it (Fig. 5C, D). Moreover, we measured the phosphorylation and total protein levels of p38 in NPCs after 30 and 60 min of treatment with IL-1β (10 ng/ml) and TAK-715(1.0 μM). Based on these data, TAK-715 inhibits NPC inflammation by targeting p38MAPK.

### TAK-715 inhibited p38MAPK pathway activation and alleviated ECM degradation in an IL-1β-induced ex vivo IVD culture model

**Fig. 6:**
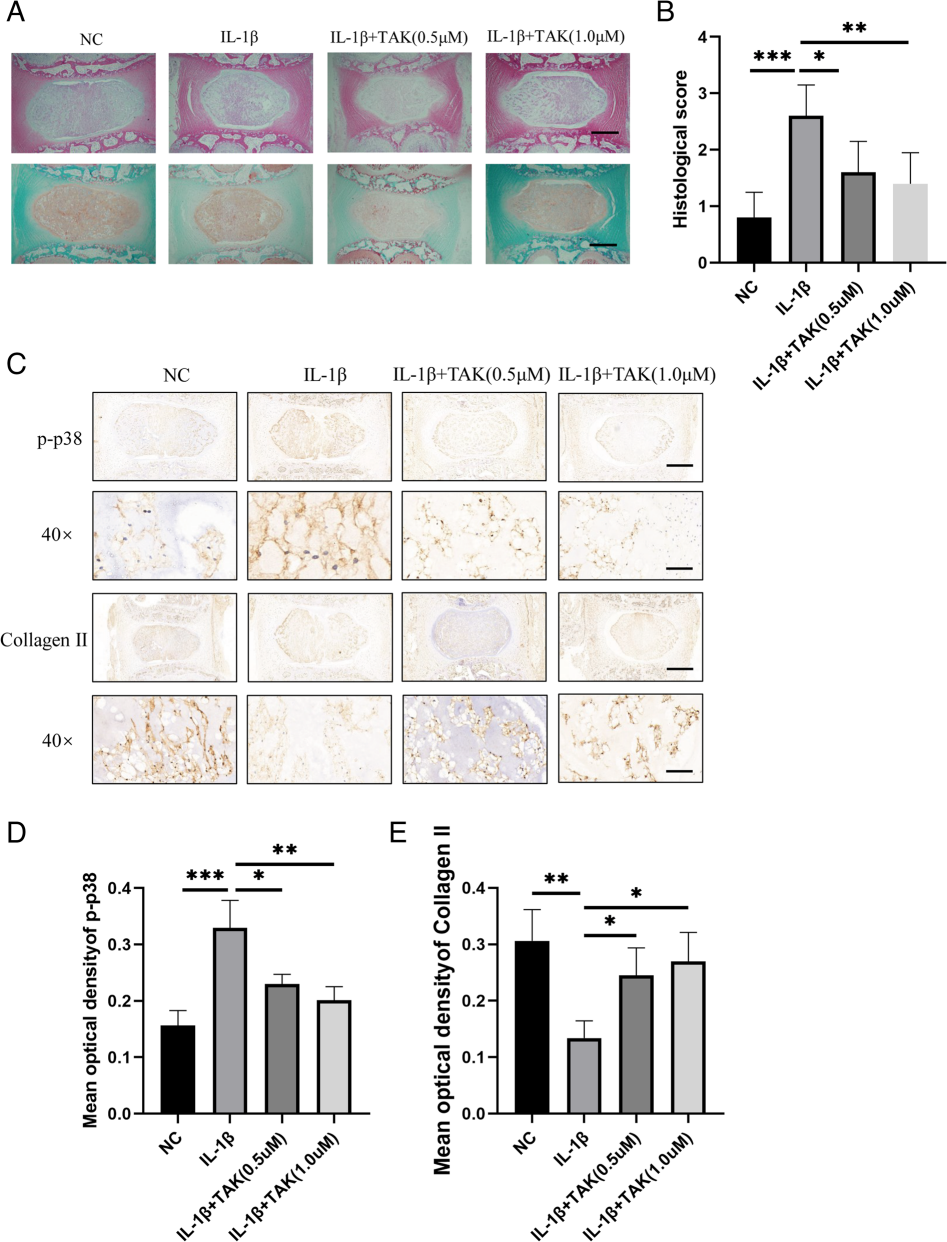
TAK-715 inhibited p38MAPK pathway activation and alleviated ECM degradation in ex vivo IVD culture model. **A** Representative HE and SO/FG staining of ex vivo IVD (scale bar:200 μm); **B** the histological grades were evaluated (*n* = 5) **C** IHC was used to determine the protein expression of p-p38 and Collagen II (upper scale bar:200 μm, lower scale bar:20 μm). **D**, **E** The protein expression of p-p38 and collagen II was quantitatively analyzed (*n* = 3). The data are presented as the mean ± SD (*P < 0.05; **P < 0.01; ***P < 0.001)

Given its protective effect in vitro on NPCs, we tested whether TAK-715 could delay the progression of ECM degeneration in an ex vivo IVD culture model. There is a decrease in nucleus pulposus areas of degenerated discs, along with ruptured fibers of the annulus fibrosus [19]. As shown in Fig. 6A, B, the HE and S–O staining of the IVD showed a decreased number of NPCs and annulus fibrosus cells after the IL-1β treatment, but TAK-715 inhibited this effect. The IHC staining showed that IL-1β increased p-p38 and inhibited collagen II, whereas TAK-715 reversed these effects (Fig. 6C-E). Ultimately, these results show that TAK-715 attenuated the degeneration of discs ex vivo.

### TAK-715 ameliorates the progression of intervertebral disc degeneration in a rat puncture model

**Fig. 7:**
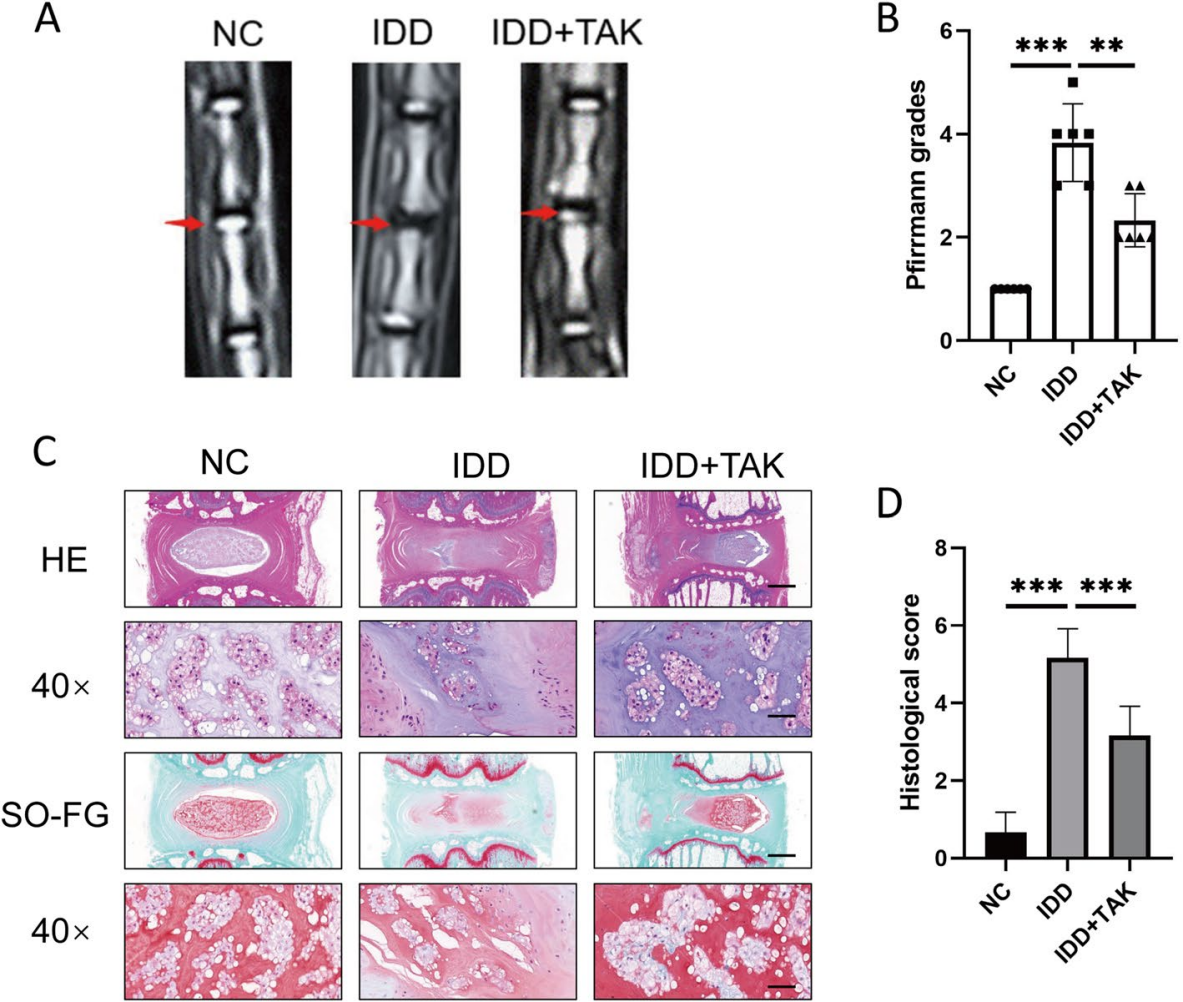
TAK-715 ameliorates the progression of intervertebral disc degeneration in a rat puncture model. **A** MRI images of rat intervertebral discs (*n* = 6). **B** Pfirrmann score of caudal MRI images of rats after surgery and injection with TAK-715; **C** HE and SO/FG staining of rat intervertebral discs (upper scale bar:200 μm, lower scale bar:20 μm). **D** The histological grades were evaluated (*n* = 6). The data are presented as the mean ± SD (*P < 0.05; **P < 0.01; ***P < 0.001)

Through the tail skin of SD rats, we stabbed the whole layer of the annulus fibrosus (AF) for one minute by using a 21 G needle. Following surgery, the TAK-715-treated rats received intradiscal injections of TAK-715, while the rats in the control and IDD groups received normal saline. At 8 weeks after the surgery, MRI images were taken, and the rats were sacrificed for a histopathological analysis. According to MRI, the signal intensity of punctured discs decreased in the IDD group, indicating that a degeneration process occurred (Fig. 7A, B). TAK-715 may be beneficial for tissues in NP based on the histological score evaluation (Fig. 7C, D). These results suggest that TAK-715 may prevent the progression of IDD in vivo by inhibiting NPC apoptosis and ECM degradation.

## Discussion

IDD is one of the main causes of low back pain, which is a serious health issue that reduces the quality of life. To date, IDD pathology and mechanisms are not completely understood. It is currently believed that IDD results from inflammatory mechanisms and an imbalance between matrix synthesis and matrix degradation [20–23]. Treatments for IDD that target inflammatory mechanisms and ECM degradation are novel approaches. To the best of our knowledge, this study is the first to demonstrate that TAK-715 inhibits IL-1β-induced NP cell apoptosis and ECM degradation. Furthermore, TAK-715 inhibits the activation of p38MAPK pathways in NPCs after IL-1β stimulation. The results of the present study indicate that TAK-715 may be a novel therapeutic agent for IDD.

TAK-715, a p38 inhibitor with oral activity, was synthesized by Japanese scholars for the first time [12]. It was found that p38 MAPK expression in degenerative nucleus pulposus was significantly increased [24]. Intervertebral disc degeneration manifests as changes in the ECM, infiltration by inflammatory cells, and apoptosis in the NP. The disks contain mainly collagens as ECM components. A healthy IVD is characterized by a relative equilibrium between the synthesis and breakdown of the ECM, and IDD is caused by the dominance of the catabolism of the ECM over the anabolism of ECM [25]. We found that TAK-715 enhanced collagen II expression and suppressed the degradation of ECM components in NPCs. Previous studies have shown that IL-1β inhibits collagen II production [26]. In addition, the degrading activity of MMPs and ADAMTSs in NPCs has been demonstrated in numerous studies [25, 27]. Based on these findings, we speculate that TAK-715 suppresses ECM degradation by inhibiting IL-1β-induced inflammation and apoptosis in NPCs. A significant decrease in inflammatory factor (COX-2 and HMGB1) expression was observed with TAK-715. According to previous research, a COX-2-mediated inflammatory response might accelerate ECM degradation by activating MMPs and ADAMTS5 [28]. HMGB1, a ubiquitous nonhistone DNA-binding protein, is a classical inflammatory molecule and mediator involved in disc degeneration [29–32]. The increasing levels of COX-2 and HMGB1 in NPCs lead to the development of IDD. The results of this research show that TAK-715 could reduce the levels of COX-2 and HMGB1 and ultimately prevent ECM degradation caused by IL-1β.

TAK-715 was further evaluated by studying the NP cell apoptosis conditions to understand how it affects disk degeneration. Researchers have found that IL-1β triggers apoptosis in NPCs [33, 34]. It has been suggested that apoptosis (also known as type 1 programmed cell death) plays an important role in disk degeneration. Hence, inhibiting apoptosis may be beneficial for stopping IDD progression. The following two interconnected pathways are involved in apoptosis: death receptors are involved in the extrinsic pathway, while mitochondria play a role in the intrinsic pathway. The biomarkers of the intrinsic pathway primarily consist of the antiapoptotic protein Bcl-2 and the proapoptotic protein Bax, which belong to the Bcl-2 family. Caspase activity is promoted by Bax; however, Bcl-2 inhibits caspase activity, thereby inhibiting apoptosis. Apoptosis is initiated and executed by the caspase family of cysteine proteases located in the cytosol. The overexpression of Bcl-2 and the knockdown of caspase3 both prevent apoptosis and degenerative changes [35, 36]. Following the treatment with TAK-715, Bcl-2 expression significantly increased, but Bax and cleaved-caspase3 expression decreased. Flow cytometry was used to further confirm the potential efficacy of TAK-715 on NP cell apoptosis. The IL-1β treatment increased apoptosis in NPCs, whereas the pretreatment with TAK-715 inhibited IL-1β-induced apoptosis. Altogether, these results indicate that TAK-715 inhibits NP cell apoptosis induced by IL-1β and that this effect may be caused by modulating Bcl-2 family members and inhibiting mitochondrial apoptosis dependent on caspases. Thus, TAK-715 alleviates intervertebral disc degeneration by inhibiting nucleus pulposus apoptosis and other diseases related to apoptosis, which may provide a therapeutic target for further study.

**Fig. 8:**
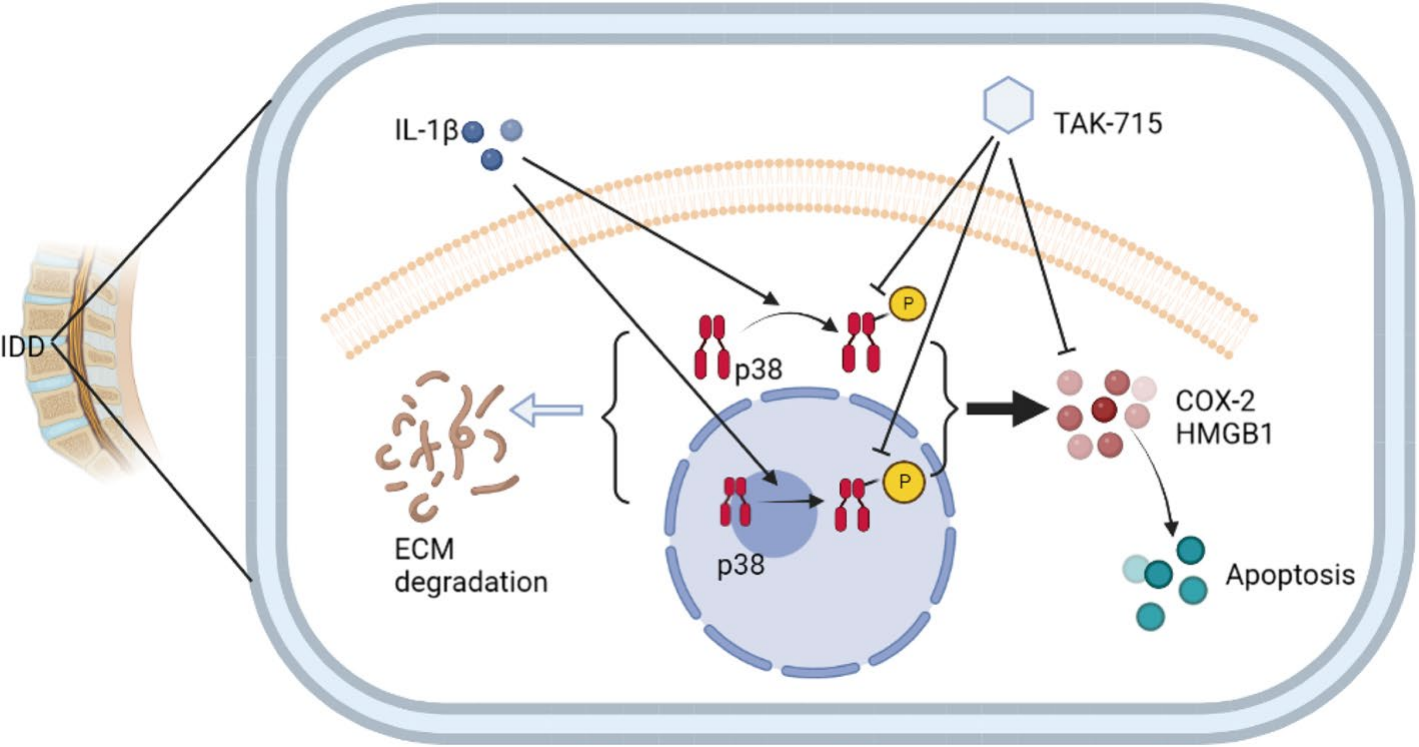
Schematic illustration of TAK-715 treatment in NPCs. TAK-715 reduced inflammation, apoptosis, and degradation of the ECM in NPCs by inhibiting the p38MAPK pathway

As a result of inflammatory factors, collagen II is degraded, and the synthesis of NPCs is inhibited, which contributes to the development of IDD. P38MAPK, a stress-activated protein kinase discovered by Brewster in 1993, triggers the inflammatory process [37]. In vitro, the degeneration of NPCs is delayed, and inflammation, pain, and disc degradation are reduced by inhibiting p38MAPK. NPCs respond less strongly to IL-1β when the p38MAPK pathway is suppressed in animal experiments, suggesting that intervertebral disc degeneration is mediated by inflammation by silencing p38MAPK. Growing evidence suggests that the p38MAPK signaling pathway is involved in inflammation, apoptosis, and IDD [38, 39]. The p38 MAPK pathway plays a major role in regulating COX-2, HMGB1 and MMP3 expression [40, 41]. In addition, the p38MAPK pathway influences the expression of collagen II, MMP9 and ADAMTS5, which are anabolic and catabolic, respectively. According to the current study, IL-1β induced the phosphorylation of p38MAPK. The TAK-715 pretreatment, however, significantly reduced IL-1β-induced p38 activation in our study. Our study found that p-p38 was highly expressed in ex vivo IVD, but collagen II was decreased. Additionally, several studies have suggested that targeting p38MAPK may be able to treat IDD [42, 43]. The overall effect mechanism of TAK-715 in NPCs was described in Fig. 8. Therefore, TAK-715 appears to be an effective, novel treatment for IDD based on these results.

## Conclusions

In summary, our findings show that TAK-715 could inhibit IL-1β-induced ECM degradation by inhibiting the p38MAPK pathway. Additionally, TAK-715 reduces the leakage of NPCs by preventing IL-1β-induced apoptosis in vitro. TAK-715 ameliorates the progression of intervertebral disc degeneration ex vivo and in vivo. As a result of these findings, TAK-715 shows promise as a potential preventative therapy for disc degeneration.

## Data Availability

The datasets used and/or analyzed in the current study are available from the corresponding author on reasonable request.

## Abbreviations

IDD: Intervertebral disc degeneration
IVD: Intervertebral disc
NPCs: Nucleus pulposus cells
NP: Nucleus pulpous
ECM: Extracellular matrix
MMP: Mitochondrial membrane potential
ADAMTS: A disintegrin and metalloproteinase with thrombospondin motifs
TAK: TAK-715
